# Microvascular Decompression of the Medulla: A Case Report and Review of the Surgical Management

**DOI:** 10.7759/cureus.91617

**Published:** 2025-09-04

**Authors:** Rachel K Kaneakua, Samir Kashyap, R Loch MacDonald

**Affiliations:** 1 Osteopathic Medicine, California Health Sciences University, Clovis, USA; 2 Neurosurgery, Riverside University Health System Medical Center, Moreno Valley, USA; 3 Neurosurgery, Community Neurosciences Institute, Community Regional Medical Center, Fresno, USA

**Keywords:** case report, literature review, medullary compression, microvascular decompression, transposition of the vertebral artery, vertebral artery compression syndrome

## Abstract

This case study and review of surgical management highlights the efficacy of microvascular decompression (MVD) with transposition of the vertebral artery in alleviating symptoms of vertebral artery compression syndrome (VACS). VACS is a rare condition in which the vertebral artery exerts pathological compression on the medulla, leading to a spectrum of potentially debilitating neurological symptoms. While microvascular decompression (MVD) is a well-documented treatment for cranial nerve compression syndromes, its application in relieving medullary compression due to VACS remains less explored.

A 49-year-old female presented with over 10 years of progressively worsening migraine headache with associated symptoms of dizziness, vertigo, blurry vision, diplopia, pulsatile tinnitus of the left ear, a sensation of a lump in her throat, hoarseness, left hemiparesis, and loss of balance. Preoperative imaging identified a tortuous, right intradural vertebral artery segment causing mass effect and deformity of the right ventral medulla. A surgical approach with MVD of the medulla and transposition of the vertebral artery led to significant symptomatic relief.

A review of surgical treatment options for VACS showed MVD with transposition of the vertebral artery resulted in improved symptoms in all cases. MVD alone showed improved symptoms postoperatively, but there was a recurrence of symptoms in some cases. Patients treated non-surgically more often showed persistent symptoms.

Advancing the management of VACS will continue to require expanding the evidence base through case reports and clinical studies, but MVD with transposition of the vertebral artery has been shown to be effective. By continuing to refine our understanding and approach, we can improve outcomes for patients afflicted by this condition.

## Introduction

Microvascular decompression (MVD) is a well-known and effective surgical treatment option for neurovascular compression syndromes such as trigeminal neuralgia, hemifacial spasm, and glossopharyngeal neuralgia [1-4]. The procedure relieves pressure on the affected cranial nerve through the insertion of padding between the compressing vessel and the nerve that frees the nerve from the offending vessel. The compression is often at the root exit zone, the transition point between the central and peripheral nervous system on the cranial nerve. Kaufmann and Prince wrote that Janetta was perhaps the first to describe this technique in 1967; he emphasized the importance of mobilizing or transposing the aberrant vessel away from the affected nerve root and changing its axis [5]. Though this technique has been recognized as a good surgical treatment option for these syndromes, the literature on microvascular decompression of the medulla is limited, likely due to lower prevalence. One of the first documented cases described in 1985 by Kim et al. reported MVD accomplished by anchoring the offending vessel to the dura, which resulted in satisfactory recovery of symptoms on a five-month follow-up with a 53-year-old man experiencing left hemiparesis due to vertebral artery compression of the left lateral medulla [6].

The medulla can be compressed by various pathological conditions or structural abnormalities such as tumors, aberrant vasculature, congenital malformations, and inflammatory conditions. This paper discusses a case of vertebral artery compression of the medulla, which has been referred to as vertebral artery compression syndrome (VACS) by Li et al. [7,8]. Clinical symptoms of VACS can arise with compression of medullary structures, such as motor and sensory pathways and/or cranial nerves at root exit zones by an aberrant vertebral artery. However, it is important to note that vertebral artery compression does not always result in VACS. The patient in this case study presented with a constellation of symptoms that can be attributed to the compression of the brainstem, as well as other symptoms that are less likely to be directly linked. Interestingly, all symptoms significantly resolved after surgery. 

## Case presentation

A 49-year-old female presented with an over-10-year history of migraine headache with aura. The headache was located in the right temporal and occipital areas and described as moderate to severe pressure, throbbing, pounding, sharp, and stabbing pain that lasted hours to days and occurred almost daily. Preceding the headache were feelings of impending doom. In addition to the headache, she noted dizziness, vertigo, blurry vision, diplopia, pulsatile tinnitus of the left ear, a sensation of a lump in her throat, hoarseness, left hemiparesis, and loss of balance. The symptoms were worse with exercise, heat, and lack of sleep, and improved with sleep, darkness, and ice. The symptoms progressively worsened and had become substantial enough for her to miss work as a massage therapist and yoga instructor.

**Figure 1:**
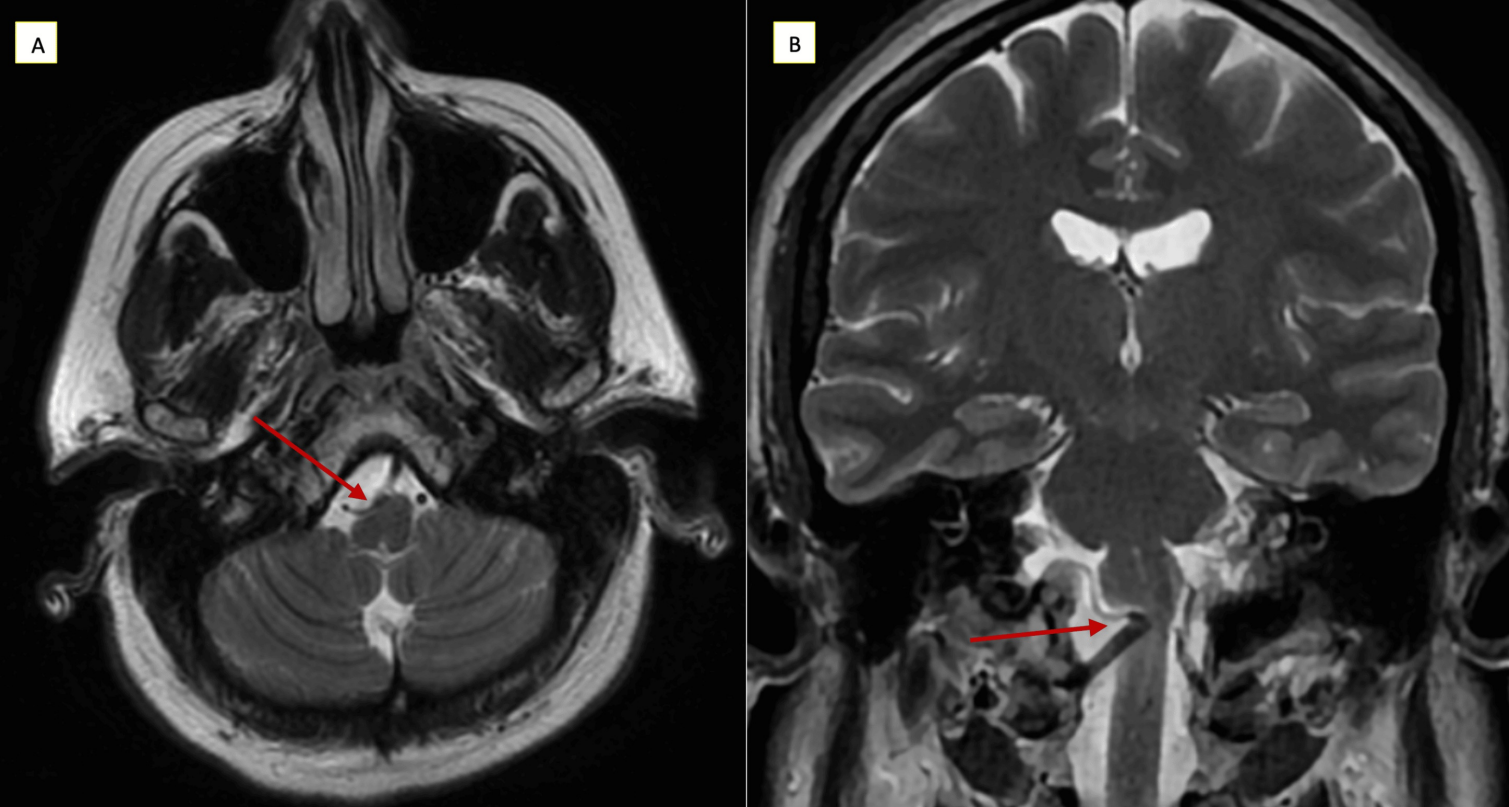
Magnetic resonance brain images A (axial view) and B (coronal view) shows lower brainstem flow void of the right vertebral artery coursing across the ventral medulla (arrows) with indentation of the right ventral lateral aspect of the medulla and mild underlying edema

Physical exam was unremarkable except for a positive Romberg test. Cranial nerves, motor strength, and sensation were intact. Upper and lower extremity reflexes were normal. Laboratory tests showed only mild anemia. Magnetic resonance imaging of the brain with and without contrast did not show evidence of a mass lesion or significant signal changes. However, there was a tortuous, right intradural vertebral artery segment with mass effect and deformity of the right ventral medulla, raising the concern for brainstem vascular compression syndrome (Figure 1). 

Nonsurgical management

This patient was treated prior to neurosurgery referral for years with rimegepant, ubrogepant, sumatriptan, butalbital/acetaminophen/caffeine, onabotulinumtoxin A, topiramate, and propranolol. Most were given based on a diagnosis of migraine, although none were of lasting or substantial benefit. The patient works as a yoga instructor and underwent multiple rounds of physical therapy and nonsurgical techniques without any improvement. She was no longer able to work regularly due to her symptoms. Given her refractory migraine headaches and superimposed symptoms, she was referred for neurosurgical evaluation for right vertebral artery compression of the medulla. 

Surgical management

**Figure 2:**
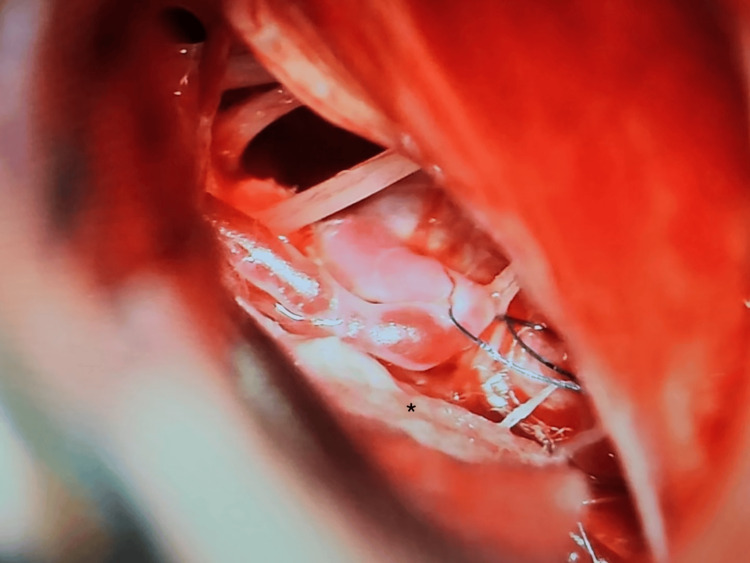
Vertebral artery being anchored to the dura using a 5-0 Prolene suture to hold it away from the brainstem * - Telfa patty overlying the brainstem

Microvascular decompression of the medulla and transposition of the vertebral artery were planned via a right retrosigmoid approach. Neuromonitoring was utilized, and baseline signals were obtained. A standard retrosigmoid craniotomy was performed. The arachnoid was released, and cranial nerves IX, X, and XI, the vertebral artery, and the posterior inferior cerebellar artery were visualized. The vertebral artery was identified, where it was compressing the medulla. Further inspection of the vertebral artery did not reveal any perforating branches from the compressing segment. An additional arachnoid was released, and a polytetrafluoroethylene pledget was placed between the vertebral artery and the medulla. Next, a polypropylene suture was used to create an anchoring stitch around the pledget on the vertebral artery and then attached to the posterior fossa dura, creating the standard sort of sling to pull the vertebral artery away from the brainstem (Figure 2). The tension on the suture was adjusted to prevent occlusion or kinking of the vertebral artery and to keep it away from the medulla. All neuromonitoring was stable. The patient was admitted to the intensive care unit for recovery.

Early postoperative course

**Figure 3:**
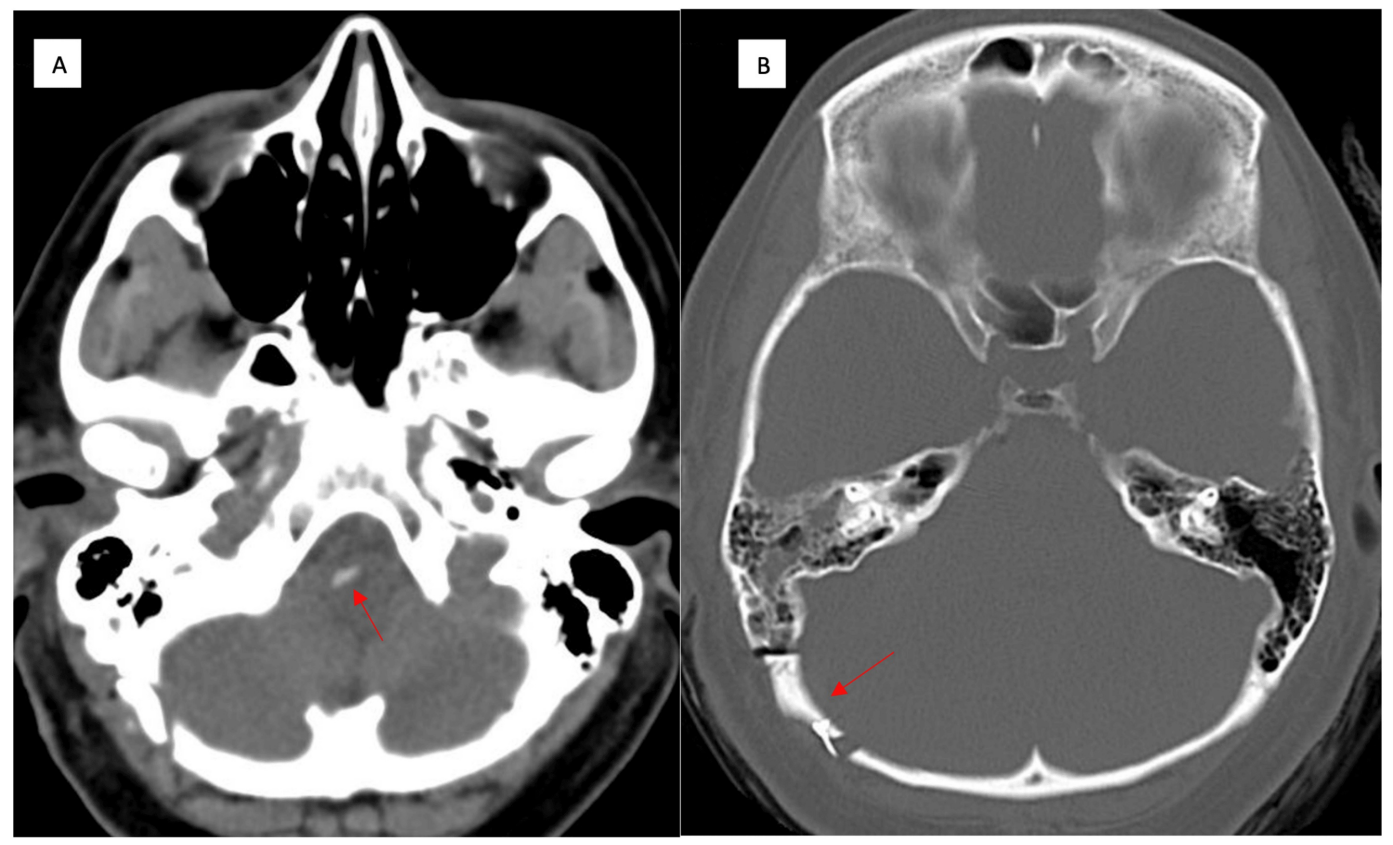
Postoperative computed tomography (CT) scan performed one day after surgery Image A axial view shows the position of the pledget next to the medulla on the right (arrow). Image B is the same CT scan with bone windowing at a more cephalad level and shows the position of the craniotomy (arrow) and opening into the mastoid air cells.

The patient had right cerebrospinal fluid otorrhea. No mastoid air cells had been entered during the surgery. Otoscopy did not show tympanic membrane perforation. Postoperative computed tomography did not show fluid in the mastoid air cells (Figure 3). The exact etiology of the fluid leakage was somewhat unclear, but it resolved after two days of lumbar cerebrospinal fluid drainage.

Follow-up

At the two-month follow-up, the patient reported that all of her preoperative symptoms had completely resolved. She no longer had any dizziness, vertigo, diplopia, dysphagia, pulsatile tinnitus, hoarseness, hemiparesis, or loss of balance. Additionally, the patient's migraine headaches were reduced to about once a month compared to almost daily. Abortive therapy for migraine headache was also significantly more effective. She was able to return to her work as a yoga instructor and massage therapist.

## Discussion

Clinical presentation of VACS is quite variable, and symptomatology can be widespread due to the nature of the posterior fossa anatomy. The varied symptoms that are usually due to other causes, including functional disorders, make a detailed history and physical in conjunction with brain imaging crucial for identifying and diagnosing VACS as a potential cause. It is important to note that not all patients with structural compression of the medulla by a dilated or tortuous vertebral artery on brain imaging are symptomatic. In fact, a significant portion of patients are asymptomatic. This is consistent with imaging studies showing that vascular compression of the trigeminal nerve in its cisternal segment is commonly asymptomatic.

VACS refers to both clinical and radiological findings explaining symptomatology after ruling out other causes such as multiple sclerosis, mass, or stroke. Clinical symptoms typically include hemiparesis, dysphonia, dysphagia, tinnitus, and gait disturbance [7-10]. If there is concomitant compression of the upper spinal cord, symptoms of cervical myelopathy can also be present. This patient had symptoms of headache, dizziness, vertigo, blurry vision, diplopia, pulsatile tinnitus of the left ear, a sensation of a lump in her throat, hoarseness, left hemiparesis, and balance issues. The patient's exertional headache is hypothesized to result from increased vertebrobasilar blood pressure or flow during exercise, exacerbating mechanical compression of the medulla by a tortuous, dilated vertebral artery. The compression of the brainstem could lead to the release of vasoactive neuropeptides, such as calcitonin gene-related peptide, contributing to headache via mechanisms similar to those observed in primary headache disorders. Additionally, compression of the right medulla could lead to it being compressed against the left vertebral artery, causing more diffuse symptoms. Compression of the anterolateral surface of the ventral medulla along the corticospinal and spinocerebellar tracts can cause hemiparesis and gait disturbance ipsilaterally or contralaterally, depending on whether the compression is above or below the pyramidal tract decussation. In this case, right-sided vertebral artery compression of the medulla above the decussation of the corticospinal tract caused left-sided hemiparesis. Compression of medullary cranial nerves at root exit zones can explain symptoms of dysphonia and dysphagia. Dizziness and tinnitus could be attributed to compression of the vestibulocochlear nuclei within the medulla, compression of the root exit zone at the vestibular cranial nerve near the cerebellopontine angle, or secondary to the headache. Given the significant improvement of all the patient's symptoms after surgery, VACS seems to be the cause.

Successful treatment options for VACS have been limited to surgical intervention, differing from other microvascular compression syndromes, which can be managed by both pharmacological and surgical approaches. Surgical treatment of trigeminal neuralgia, glossopharyngeal neuralgia, and hemifacial spasm with MVD seems to be very effective. However, the anatomy of the vertebral artery poses extra challenges that make MVD a less optimal choice because of the need to manipulate the brainstem, maneuver around multiple critical and sensitive lower cranial nerves and brainstem perforating arteries, and create a surgical construct that will prevent the vertebral artery from reverting back to its original position. One report that looked at 33 cases of VACS found that repositioning or moving the vertebral artery away from the brainstem seemed better at alleviating symptoms than simply putting a pledget or padding between the artery and the brainstem [8]. This is likely due to the nature of the pulsations, dimensions, and stiffness of the vertebral artery, which requires repositioning over decompression. However, vertebral artery repositioning is a more risky procedure that needs to be considered on a case-by-case basis.

We reviewed case reports of patients with VACS (Table 1). MVD with transposition of the vertebral artery resulted in improved symptoms after surgery in all cases, including the present case (n=25). MVD alone showed improved symptoms after surgery in most cases (n=14), 93% of cases showed improvement, with one patient having a recurrence of symptoms at one-year follow-up. Patients treated non-surgically more often showed persistent symptoms (n=26), and 35% of cases showed improvement with various conservative treatment options. There is limited long-term follow-up on patients treated both surgically and non-surgically, which makes it difficult to say whether or not symptoms recurred past the follow-up appointment stated in each case. It is also difficult to know the extent of improvement among patients, given that it was not clearly defined in many of the case studies that were reviewed. 

**Table 1:** Review of case reports of vertebral artery compression syndrome of the medulla CN - cranial nerve; CSA - central sleep apnea; DCML - dorsal column medial lemniscus; LE - lower extremity; LOC - loss of consciousness; N/A - not available; OSA - obstructive sleep apnea; UE - upper extremity; VA - vertebral artery

Author	Age/gender	Imaging	Symptoms	Treatment	Outcome, follow-up
Kim et al., 1985 [6]	53M	Thick, torturous left VA	Left hemiparesis	MVD and anchoring to the dura mater	Gradual improvement, 5 months
Li et al., 2019 [7]	59F	Left VA compression	Left leg hemiparesis, hyperreflexia	None	Progressive, N/A
Li et al., 2019 [7]	82M	Right VA compression	Right leg hemiparesis	None	Persistent, N/A
Li et al., 2019 [7]	52F	Left VA compression	Left hemiparesis	None	Persistent, N/A
Li et al., 2019 [7]	51F	Left VA compression	Left hemiparesis	None	Persistent, N/A
Li et al., 2019 [7]	61M	Right VA compression	Left hemiparesis	Nonspecific	Persistent, N/A
Li et al., 2019 [7]	41M	Left VA compression	Dysphagia, gait disturbance	Nonspecific	Persistent, N/A
Li et al., 2019 [7]	72F	Left VA compression	Dysarthria, ataxia	None	Persistent, N/A
Li et al., 2019 [7]	71F	Left VA compression	Dysarthria, imbalance	Aspirin	Improved, N/A
Li et al., 2019 [7]	66F	Left VA compression	Vertigo, imbalance	Aspirin	Improved, N/A
Li et al., 2019 [7]	73F	Left VA compression	Vertigo, imbalance	Aspirin	Improved, N/A
Li et al., 2019 [7]	74M	Left VA compression	Left leg paresis	Physiotherapy	Persistent, N/A
Lombarski et al., 2018 [8]	36M	Tortuous right VA	Left hemiparesis and loss of spinothalamic tract	MVD with Gore-Tex implant	Improved, 1 year
Tomasello et al., 2005 [11]	63M	Right ectatic VA	Dysphonia, dysphagia	MVD and anchoring to the dura mater	Improved, 1 year
Tomasello et al., 2005 [11]	58M	Left ectatic VA	Dysphagia, weakness of bilateral legs	MVD and anchoring to the dura mater	Improved, 1 year
Tomasello et al., 2005 [11]	55F	Right ectatic VA	Dysphagia and weakness of right arm and leg	MVD and anchoring to the dura mater	Improved, 1 year
Tomasello et al., 2005 [11]	67F	Right ectatic VA	Weakness of upper and lower extremities	MVD and anchoring to the dura mater	Improved, 1 year
Tomasello et al., 2005 [11]	73M	Left ectatic VA	Weakness of upper and lower extremities	MVD and anchoring to the dura mater	Partial improvement, 1 year
Hongo et al., 1999 [12]	47M	Tortuous left VA	Vertigo, gait disturbance	MVD and VA anchoring to dura with Gore-Tex	Improved, 3 months
Hongo et al., 1999 [12]	36M	Tortuous right VA	Right hemiparesis	MVD and VA anchoring to dura with Teflon felt	Gradual improvement, 9
Vincentelli et al., 1991 [13]	38M	Ectatic right VA	Vertigo, left hemiparesis	MVD and anchoring to dura with Gore-Tex	Improved, 2 years
Maruyama et al., 1989 [14]	30M	Elongated VA crossing midline medulla	Right hemiparesis, spastic gait	MVD	Improved then recurrence 1 year later
Savitz et al., 2006 [15]	32F	Tortuous left VA	Hoarseness, dysphagia	MVD	Improved, unknown
Savitz et al., 2006 [15]	71M	Tortuous left VA	Tinnitus	Aspirin	No improvement
Savitz et al., 2006 [15]	79M	Tortuous left VA	Left leg weakness	Aspirin and dipyridamole	Improved, 1 year
Savitz et al., 2006 [15]	35F	Tortuous VA	Headaches	Analgesics	Improved, 1 year
Savitz et al., 2006 [15]	68M	Tortuous left VA	Balance issues	Warfarin sodium	Improved, 1 year
Savitz et al., 2006 [15]	63F	Tortuous right VA	Left hemiparesis	Aspirin	Moderate improvement, 3 years
Savitz et al., 2006 [15]	34F	Tortuous left VA	Balance issues, aural fullness, headache	Aspirin	No improvement
Savitz et al., 2006 [15]	63F	Tortuous left VA	Left hemiparesis	Aspirin	Improved, 4 years
Ghorbani et al., 2023 [16]	43M	Tortuous left VA	Hypersomnia, central sleep apnea	MVD with synthetic Teflon patch	Improved, 3 months sleep apnea
Kutty et al., 2020 [17]	57M	Ectatic left VA	Dizziness, up and down beat nystagmus	MVD with Teflon sponge	Gradual improvement, N/A
Hoffman & Stiller, 1995 [18]	62M	Tortuous basilar looping left VA	OSA	MVD with Teflon sponge	Improved, 3 months
Ascanio et al., 2017 [19]	57M	Ectatic left VA	Left hemiparesis	MVD and sling repositioning to dura	Improved, 3 months
Kobayashi et al., 1992 [20]	39F	Bilateral VA compression, Chiari malformation	Gait disturbance, LE paresis	MVD and anchoring to dura with	Improved postoperatively, N/A
Hongo et al., 1993 [21]	30M	Elongated left VA	Paresis of LE, right UE	MVD	Improved postoperatively, recurred within 5.5 years
Murata et al., 1995 [22]	58F	Tortuous bilateral VA	Quadriparesis	MVD	Improved postoperatively, N/A
Salvi et al., 2000 [23]	54F	Tortuous left VA	Hyperekplexia, spastic paresis	MVD	Improved postoperatively, N/A
Salvi et al., 2000 [23]	74F	Ectatic left VA	Right hemiparesis and loss of vibration sense	Refused surgery	N/A
Meyer et al., 2000 [24]	47M	Ectatic left VA	Palatal myoclonus	MVD	Improved postoperatively, N/A
Koyama, 2001 [25]	51M	Bilateral VA compression	Transient paresis, LOC	MVD and VA anchoring to dura with Gore-Tex	Improved postoperatively, N/A
Takano et al., 2001 [26]	53F	Elongated and tortuous left VAtortuous	Headache, right hemiparesis, hoarseness, hypertension	MVD	Gradual improvement, 2 months
Ubogu et al., 2002 [27]	70M	Ectatic left VA	Quadriparesis, CN deficits	MVD and VA anchoring to dura with Gore-Tex sling	Moderate improvement, 6 months
Vanikieti et al., 2016 [28]	50F	Ectatic left VA	Palatal tremor, oscillopsia	MVD	Unchanged, 2 months
Sadashiva et al., 2016 [29]	36M	Tortuous right VA	Left hemiparesis	MVD with VA anchoring to dura	Improved, 14 months
Sadashiva et al., 2016 [29]	55M	Bilateral ectatic VA	Urinary incontinence, right hemiparesis	Refused surgery	No improvement, 58 months
Sadashiva et al., 2016 [29]	63M	Ectatic left VA	Quadriparesis	Refused surgery	No improvement, 50 months
Rahimi et al., 2008 [30]	55M	Bilateral VA compression	Dysphagia, dysarthria	MVD with VA anchoring to dura with Gore-Tex sheet	Improved postoperatively, N/A
Pereira-Filho et al., 2008 [31]	60M	Tortuous left VA	Left hypereflexia, dysarthria	MVD with VA anchoring to dura	Improved, 6 months
Hanggi & Steiger, 2009 [32]	24M	Elongated right VA	Headache, CN 5 V3 neuralgia, uvula deviation	MVD with VA anchoring to dura with Gore-Tex sheet	Improved, 18 months
Hanggi & Steiger, 2009 [32]	25M	Elongated left VA	Headache	MVD with VA anchoring to dura with Gore-Tex sheet	Improved, 18 months
Hung & Shen, 2011 [33]	54M	Ectatic left VA	Dysarthria, dysmetria, dysdiadochokinesia	MVD with VA anchoring to dura with silk thread	Gradual improvement, 6 months
Kamada et al., 2013 [34]	58M	Tortuous bilateral VA	Spastic paraparesis	MVD right VA	Gradual improvement, N/A
Nakahara et al., 2014 [35]	71F	Right VA compression	Dysphagia, dysarthria	MVD with VA anchoring to dura with Gore-Tex	Improved, 2 months
Nakahara et al., 2014 [35]	72F	Left VA compression	Dyspnea requiring intubation	MVD with VA anchoring to dura	Gradual improvement, 2 months
Nakahara et al., 2014 [35]	71F	Bilateral VA compression	Dysphagia, hoarseness	MVD with right VA anchoring to dura	Gradual improvement, 2 months
Gorton et al., 2015 [36]	69F	Tortuous left VA	Intractable nausea, vomiting, dizziness, diplopia	MVD with anchoring to dura with sling	Improved, 2 years
Ren et al., 2017 [37]	67F	Tortuous and ectatic left VA	Right hemiparesis and spasms	MVD	Improved, 1 year left VA
Cai et al., 2022 [38]	65M	Tortuous left VA	Left limbs spasticity	Refused surgery, antispasmodics	Improved, 6 months
Seo et al., 2019 [39]	41F	Tortuous left VA	Gait disturbance, DCML dysfunction on the right	Refused surgery	N/A
Miyazaki et al., 1991 [40]	5M	Looping left VA	CSA	Refused surgery, acetazolamide	No improvement
Younus et al., 2021 [41]	80M	Ectatic right VA	Dysphagia, dysphonia	Surgery contraindicated	Patient deceased, 3 months - pneumonia
Dembo & Tanahashi, 2013 [42]	55M	Tortuous and ectatic left VA	Vertigo, nausea, vomiting, hypertension, gait imbalance, left hemiparesis	Antihypertensives	Improved, 2 months
Rafaelyan & Svistov, 2022 [43]	28M	Ectatic right VA	Dysarthria	MVD with anchoring to dura with Teflon cuff	Improved, 3 months

This case study adds to a small but growing number of reports of people undergoing surgical management of VACS. Favorable outcomes were achieved in a majority of patients using this approach. However, the symptoms attributed to VACS are quite disparate and often a part of functional-type disorders. Additionally, vertebral artery compression of the brainstem can be asymptomatic and therefore, it is questioned whether improvement after surgery for VACS is based on some concrete pathophysiological thing or on a functional basis. Of course, the same can be said about trigeminal neuralgia, although the striking effect of MVD in those cases is difficult to miss.

Alternative approaches are being explored, such as the use of flow diversion. This may provide a minimally invasive solution for changing the trajectory of the vertebral artery and reducing its pulsatile impact on the medulla. Mohammad et al. presented a case of a patient with sensorineural hearing loss and disequilibrium secondary to compression of the brainstem from a fusiform basilar artery aneurysm that experienced complete resolution of clinical symptoms after placement of a flow diverter [44]. Flow diverter therapy was also successful in the treatment of eosinophilic granulomatosis with polyangiitis-induced vertebral artery aneurysmal compression of the medulla after one year of failed immunosuppressive therapy [45]. This highlights the potential use of a flow diverter to relieve neurovascular compression syndromes.

## Conclusions

This case highlights the efficacy of MVD with transposition of the vertebral artery in alleviating symptoms of medullary compression and reviews surgical treatment options. MVD with transposition of the vertebral artery resulted in improved symptoms in the small but growing number of surgically treated cases of VACS compared to MVD alone. Advancing the management of VACS will continue to require expanding the evidence base through case reports and clinical studies. By continuing to refine our understanding and approach, we can improve outcomes for patients afflicted by this condition. Further research will be essential in identifying patterns, optimizing treatment strategies, and ultimately enhancing patient care. As awareness grows, the potential to develop standardized guidelines can aid in diagnosis and intervention.
